# Radiometals in Diagnosis and Therapy

**DOI:** 10.1155/MBD.1997.153

**Published:** 1997

**Authors:** James F. Burke

**Affiliations:** Healthcare Research and Development Amersham International plc Amersham Laboratories Bucks Amersham HP7 9LL UK

## Abstract

Radiometals are the mainstay of both diagnostic and therapeutic nuclear medicine because the choice of radionuclide is primarily dictated by nuclear decay characteristics rather than chemistry. ^99m^Tc is the most frequently used diagnostic radionuclide and requires coordination in a diversity of chemical disguises to permit imaging of a variety of tissues and disease states. Therapeutic nuclear medicine is less advanced but can provide significant benefits provided the radionuclide is accurately targetted.


### RADIOMETALS IN DIAGNOSIS AND THERAPY

James F. Burke

Healthcare Research and Development, Amersham International plc, Amersham Laboratories, Amersham, Bucks HP7 9LL, UK

**Abstract**
Radiometals are the mainstay of both diagnostic and therapeutic nuclear medicine because the choice of radionuclide is primarily dictated by nuclear decay characteristics rather than chemistry. $^{99m}$Tc is the most frequently used diagnostic radionuclide and requires coordination in a diversity of chemical disguises to permit imaging of a variety of tissues and disease states. Therapeutic nuclear medicine is less advanced but can provide significant benefits provided the radionuclide is accurately targetted.

### Introduction

Radiopharmaceuticals are radioactive drugs which can have either diagnostic or therapeutic applications. In diagnostic nuclear medicine, the radionuclide is used as a tracer to provide functional information about the tissue under investigation. The very short-lived positron-emitting radionuclides such as $^{11}$C, $^{13}$N, $^{15}$O and $^{18}$F are used for diagnosis in hospitals with access to highly specialised and expensive positron emission tomography (PET) facilities. However, most hospital nuclear medicine departments are not equipped for such work and need to use radionuclides suitable for single photon emission tomography (SPET). The nuclear properties required for such radionuclides are:

1. A half-life long enough both to make it readily available in the hospital and to allow it to be distributed *in vivo* according to the physiological function being investigated, but not so long as to give the patient more radiation dose than is justified by the requirements of the study. These needs frequently dictate a half-life of 2-3 days.
2. A decay which gives rise to penetrating γ-emissions to allow the distribution of the radionuclide to be measured, but is not accompanied by damaging, non-penetrating particulate radiations such as α- or β-particles.

In contrast, therapeutic radionuclides should have particulate radiations with an *in vivo* range sufficient to destroy the target tissue but not so long as to cause collateral damage and have little or no accompanying γ-emissions which could cause unnecessary radiation exposure to both the patient and hospital staff. Again, the half-life needs to be sufficiently long to allow availability to the hospital and *in vivo* distribution but is otherwise dictated by whether the radiation dose is to be delivered at a high dose rate over a short time or at a lower dose rate over an extended period. The use of radionuclides in therapy is less advanced than in diagnosis because destructive doses require much more accurate targeting than tracer doses.

The nuclear requirements for either diagnosis or therapy are seldom met by the radionuclides of the biologically ubiquitous elements such as C, H, N, O *etc.* but oblige the use of biologically unimportant or irrelevant elements, almost all of which are metallic. Although some radiometals will target a particular tissue when administered as a simple metal salt, it is frequently the case that the radiopharmaceutical chemist is required to coordinate the metal in a chemical Trojan Horse to cause it to be delivered to its biological target. The role of coordination chemistry in nuclear medicine has been reviewed by Jurisson *et al*[1]. This paper will illustrate the use of radiometals in nuclear medicine by discussing the use of the $^{99m}$Tc coordination compounds $^{99m}$Tc-HM-PAO for imaging cerebral blood flow and $^{99m}$Tc-HL91 under development for imaging hypoxic tissue and the application of $^{89}$Sr as the chloride salt for the treatment of metastatic bone pain.

### The Role of $^{99m}$Tc in Diagnostic Nuclear Medicine

Tc is a second row transition metal which did not occur on Earth before its artefactual production, mainly as a product of $^{235}$U fission. $^{99m}$Tc has nuclear decay characteristics which make it the most frequently used diagnostic radionuclide. It decays with a 6.02 hour half-life by isomeric transition to $^{99}$Tc. The decay is accompanied by the emission of a 140 keV γ-ray (89%) which is near-ideal for imaging with a modern γ-camera. Although $^{99}$Tc is itself radioactive (decaying by β-emission with a half-life of $2.1\times10^5$ years), the quantities produced by the decay of $^{99m}$Tc radiopharmaceuticals are not usually considered hazardous - the ratio of $^{99}$Tc radioactivity to originating $^{99m}$Tc radioactivity is the ratio of the half-lives, ~1 to $3\times10^8$. The short half-life of $^{99m}$Tc is long enough to be suitable for most nuclear medicine procedures but usually precludes convenient distribution from manufacturer to hospital radiopharmacy. However, $^{99m}Tc$ is produced by the decay of 66-hour half-life $^{99}Mo$. Distribution is achieved by the supply of commercially produced $^{99}Mo/^{99m}Tc$ generators. These contain an alumina chromatography column on which $^{99}Mo$ has been adsorbed from acidified molybdate solution. The $^{99m}Tc$ is extracted as $^{99m}TcO_4^-$ by elution with 0.9% NaCl to produce a sterile solution which is isotonic with blood and ready for injection. The chromatography column is surrounded by a lead or depleted uranium shield. As the $^{99m}Tc$ is continuously produced by the $^{99}Mo$ decay, the generator can be eluted one or more times a day. The decay and regrowth characteristics are illustrated in Figure 1 for a variety of elution times. The calculation of these curves is described in most radiochemistry or nuclear chemistry textbooks (e.g. reference 2).

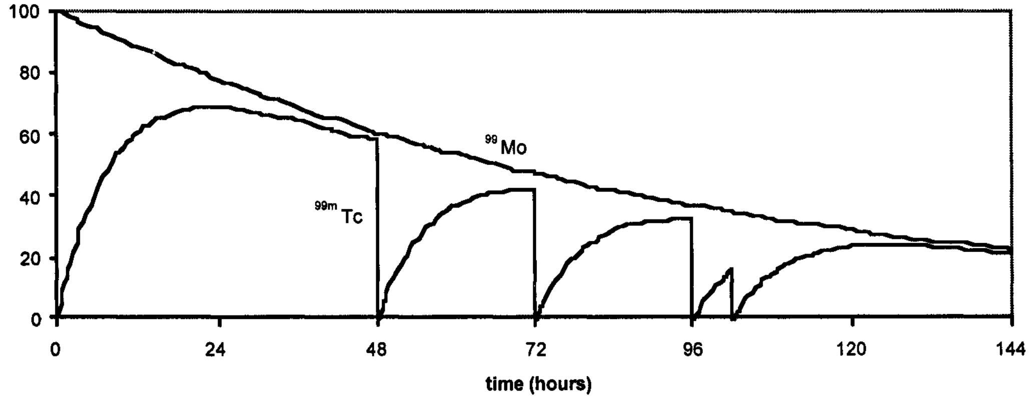

Figure 1: $^{99}Mo/^{99m}Tc$ generator elution and regrowth.

It is seen that the generator system allows preparation of $^{99m}TcO_4^-$ once or twice a day with near-optimal efficiency of $^{99m}Tc$ use. Although $^{99m}TcO_4^-$ has nuclear medicine uses in its own right, such as thyroid imaging, the majority of applications for $^{99m}Tc$ require it to be in other chemical forms. Tc exhibits a range of oxidation states from -1 to +7. The Tc in $^{99m}TcO_4^-$ is in oxidation state +7 and requires reduction and coordination with a suitable ligand system to provide an appropriately targeted radiopharmaceutical. Most $^{99m}Tc$ radiopharmaceuticals are produced from $^{99m}TcO_4^-$ with the aid of commercially produced kits, either in the hospital or at a centralised radiopharmacy supplying many hospitals in a region. The simplest and most convenient technetium kits consist of a sterile vial containing a freeze-driedmixture of a reducing agent (usually a salt of Sn(II)), a complexing agent and perhaps some excipients such as a filler or a buffer. On addition of generator eluate and any required 0.9% NaCl diluent, the $^{99m}TcO_4^-$ reacts with the kit ingredients to produce a finished radiopharmaceutical in just a few minutes.

**The Use of $^{99m}$Tc-HM-PAO for Imaging Brain Blood Flow**

Imaging of brain blood flow is used for the diagnosis and management of patients with a wide variety of neurological and psychiatric conditions such as stroke, epilepsy, trauma, depression and dementias of various origins. In the 1980's, the discovery of $^{99m}Tc$-based agents for imaging brain blood flow was an important goal for radiopharmaceutical research. The need for the molecule to cross the intact blood-brain barrier (BBB) dictated that it should be electrically neutral, of low molecular mass (<500 daltons) and lipophilic[3]. Several $^{99m}Tc$ complexes were identified as potential brain blood flow agents but only $^{99m}Tc$-HM-PAO (Ceretec™, Amersham International) and $^{99m}Tc$-ECD have progressed to routine clinical use[4]. The development of $^{99m}Tc$-HM-PAO stemmed from the discovery that the $^{99m}Tc$ complex of PnAO (Figure 2) possesses the properties required to cross the BBB[5] and shows transient flow-related brain uptake in humans[6]. It is formed by reacting $^{99m}TcO_4^-$ with Sn(II) and PnAO to cause reduction of the Tc from oxidation state +7 to +5 and complexation by the PnAO ligand to give a lipophilic neutral molecule. The Tc-PnAO did not persist in the brain for long enough to permit imaging using conventional SPET equipment. Synthesis of a range of PnAO derivatives led to the discovery of $^{99m}Tc$-HM-PAO[3]. This molecule is unstable in aqueous solution, reacting to produce a poorly characterised hydrophilic $^{99m}Tc$ complex. Its *in vivo* behaviour appears to be similar except that the intracellular rate of conversion to a hydrophilic complex is sufficiently fast to cause ~50% of the $^{99m}Tc$ that enters brain to be trapped[7]. The greatly enhanced rate of conversion *in vivo* appears to be due to reaction with intracellular glutathione. The trapping arises because the conversion product is too hydrophilic to cross the BBB and so is unable to diffuse out of the brain. The resultant distribution of $^{99m}Tc$ within the brain gives a snapshot of blood flow at the time of injection. Figure 3 illustrates this with planar (as opposed to tomographically reconstructed SPET) scans of normal and brain-dead subjects - the radioactivity is only deposited in viable tissue. Figure 4 shows SPET images of a trauma patient 2 weeks after injury and following recovery. The scans give detailed information about the progression of brain function.

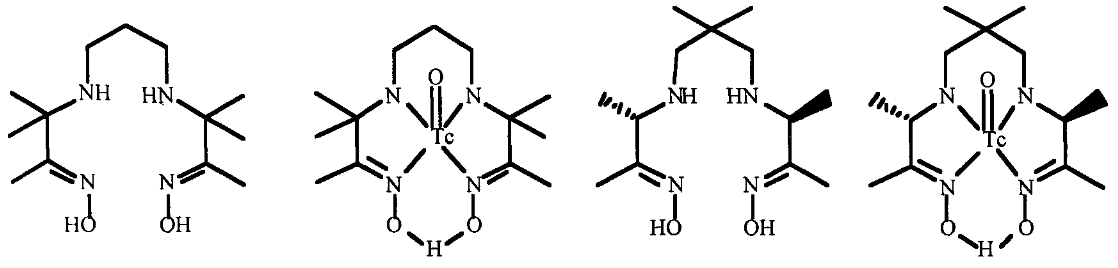

Figure 2: Amine oxime ligands and complexes. The HM-PAO used is the *d,l*-racemate.

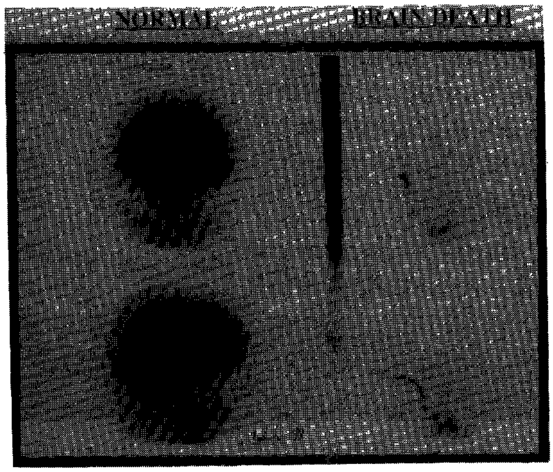

Figure 3: Planar $^{99m}$Tc-HM-PAO brain scans of normal and brain-dead subjects. The dense radioactivity distribution in the normal subject is consistent with the regional distribution of brain blood flow. In the brain-dead subject, radioactivity is concentrated in the catheter used for administering the injection but is absent from the brain. $^{99m}$Tc-HM-PAO is normally administered by intravenous injection in the arm. (Images reproduced courtesy of Dr D. C. Costa[8], University College London Medical School, London, UK).

**The Development of $^{99m}$Tc-HL91 for Imaging Hypoxic Tissue**

The imaging of hypoxic (under-oxygenated) tissue is of potential importance because it may allow the identification and management of tumours resistant to radiotherapy or of at risk myocardium (heart muscle) in heart patients. A possible targeting mechanism is the intracellular reduction of nitroimidazoles[9] (Figure 5). The *in vivo* reduction of nitroimidazoles is mediated by cytosolic or mitochondrial enzymes but, in normoxic (normally oxygenated) cells, the first step is reversed due to reaction with oxygen (the "futile cycle"). In hypoxic cells, this reversal does not take place and products which may be unable to diffuse out of the cell occur.

The first $^{99m}$Tc agent to employ this trapping mechanism was BMS-181321[10] (Figure 6). Inspection of Figure 2 shows this is formed from a nitroimidazole derivative of PnAO. While BMS-181321 exhibits uptake and retention in hypoxic tissue, its very high lipophilicity results in poor clearance from background tissue, especially the liver[11]. A Tc complex of HL91 (also known as $Bn(AO)_2$) has been described by Jurisson *et al*[12]. That work identified the complex as containing a *trans*-$TcO_2$ core. However, subsequent studies suggest a variety of complexes can be formed, depending on the preparation route[13]. Work at Amersham International identified the $^{99m}$Tc complex formed in aqueous solution from the HL91 nitroimidazole derivative, HL91M as a useful lead compound[14]. However, negative control experiments showed the analogous $^{99m}$Tc complex of the underivatised HL91 ligand gave more selective retention in hypoxic tissue[14]. The complex has subsequently been shown to be reducible under conditions similar to those required to reduce nitroimidazoles[13]. $^{99m}$Tc-HL91 is still undergoing development as a hypoxia imaging agent. Figure 7 shows scans of a head tumour comparing a PET metabolism image ($^{18}$F-deoxyglucose, $^{18}$FDG) with a $^{99m}$Tc-HL91 SPET image - this is one of the first patient studies with $^{99m}$Tc-HL91.

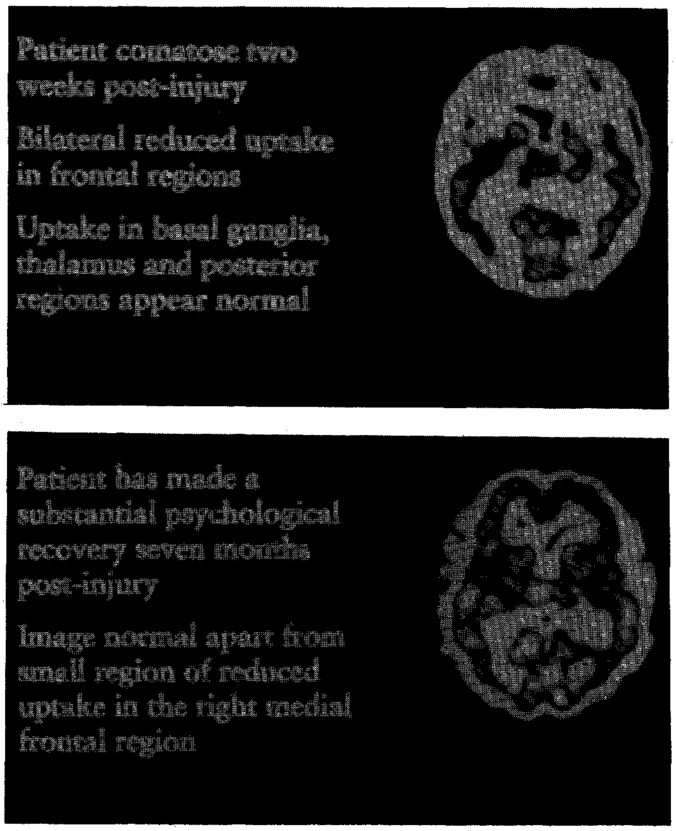

Figures 4: $^{99m}$Tc-HM-PAO brain SPET scans of an assault victim showing very low $^{99m}$Tc deposition in the frontal lobes 2 weeks after the injury and a near-normal distribution following recovery. (Images reproduced courtesy of Dr D J Wyper, Southern General Hospital, Glasgow, UK).

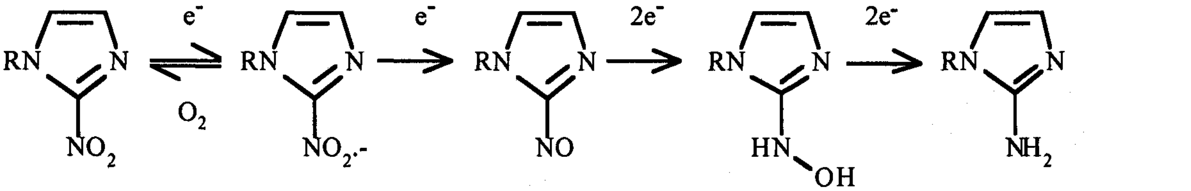

Figure 5: Intracellular reduction of nitroimidazoles.

**The Use of $^{89}$Sr-SrCl$_2$ for the Treatment of Metastatic Bone Pain**

Prostate cancer is the second most common male cancer in many Western countries. Most cases are detected too late for curative treatment. Metastases (secondary cancers) usually appear in bone. Bone pain is a common problem which is often difficult to control effectively[15]. Management of bone pain often progresses through the following stages: hormone treatment; chemotherapy and/or local radiotherapy and/or analgesics; hemibody radiotherapy; narcotics. Once these measures have failed as the disease progresses, the quality of life of such patients is frequently poor. At this stage, the use of a therapeutic radiopharmaceutical that concentrates at the sites of lesions to provide *in situ* radiotherapy can be an attractive option.

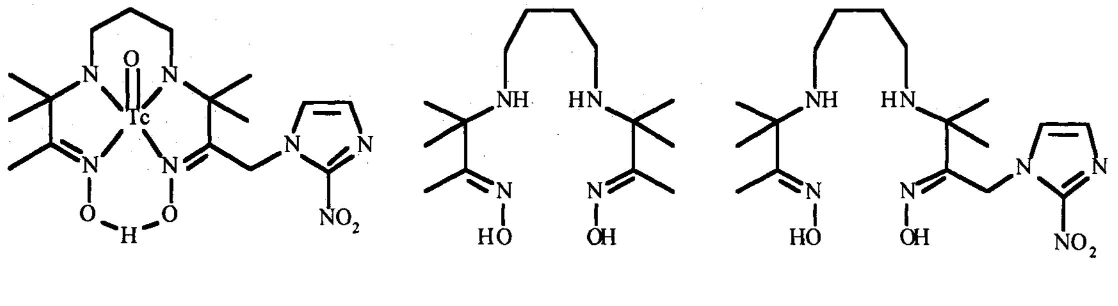

**BMS-181321** **HL91** **HL91M**

Figure 6: Amine oxime derivatives developed for imaging hypoxia.

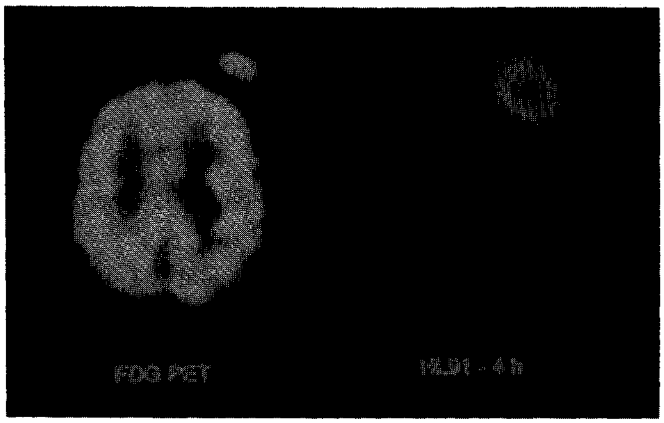

Figure 7: Tomographically reconstructed images of a transaxial section of the head of a patient with a head tumour - a lymphoma arising either from the skull or the soft tissue overlying the skull. The tumour is in the left frontal region but the patient's left and right are transposed in the images. Left image: $^{18}$F-deoxyglucose PET scan showing normal glucose metabolism in the brain and a region of high glucose metabolism corresponding to the tumour. Right image: $^{99m}$Tc-HL91 SPET scan showing intense $^{99m}$Tc deposition in the tumour but very little radioactivity elsewhere. (Images reproduced courtesy of Dr G Cook, Guy's Hospital, London, UK).

$Sr^{2+}$ can behave as an analogue of $Ca^{2+}$ (both metals are in group IIA of the periodic table; the ionic radii (2+) are 11.3 nm and 9.9 nm respectively). $^{89}$Sr decays by emission of a 1.49 MeV (maximum energy) β-particle with a half-life of 50.5 days. The β-emission has a range in bone of 3.5 mm. There are no accompanying γ-emissions. Tracer studies with the γ-emitting nuclide $^{85}$Sr show Sr administered intravenously as a solution of $SrCl_2$ is taken up in bone, concentrating at sites of high bone turnover[16]. The $^{85}$Sr washed out of normal bone but was retained at metastatic sites. $^{85}$Sr not incorporated in bone was excreted in the urine. Whole body retention of $^{85}$Sr at 3 months following injection varied from 88% in a patient with complete skeletal involvement to 11% in light disease. Consequently, $^{89}SrCl_2$ (Metastron™, Amersham International) may be administered by intravenous injection and concentrate in fast-growing regions of bone, such as metastases, destroying the metastatic tissue and causing relief of pain. Studies to establish the optimum dosage were conducted in 112 patients[17]. There was a positive dose-pain relief response relationship which rose to a plateau at 1.5 MBq/kg. The major side-effects were depression of platelets and leukocytes. At a dose of 150 MBq (~2.15 MBq/kg), the three-month post treatment levels had dropped ~30% for platelets and ~20% for leukocytes. This was adopted as the standard dose to allow for patient variation, whilst ensuring side-effects were kept below unacceptable levels. A double-blind study was conducted to compare patient response to 150 MBq $^{89}$Sr with response to placebo[18]. Patients used diaries to record their symptoms and intake of analgesic. Assessment also took into account the level of pain, appetite, mood and sleep pattern. By the time final assessments were available from 26 patients, the study was terminated because $^{89}$Sr exhibited a clear superiority and it became unethical to continue administering placebo. The results are shown in Figure 8. One third of patients showed a dramatic response to $^{89}$Sr, whereas none showed such a response to placebo.

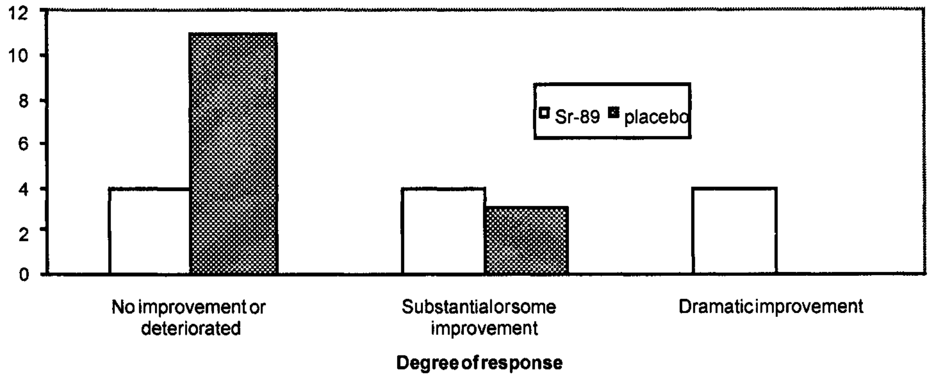

Figure 8: Assessment of response of patients to $^{89}$Sr (n=12) and placebo (n=14).

### Conclusions

Radiometals are the mainstay of both diagnostic and therapeutic nuclear medicine because the choice of radionuclide is primarily dictated by nuclear decay characteristics rather than chemistry. Although some metals are usefully targeted as simple salts, most uses require the application of carefully researched coordination chemistry to ensure delivery of the radionuclide to the target tissue.

### Acknowledgement

The author thanks Amersham International for permission to publish this paper.

### References

1. S. Jurisson, D. Berning, Wei Jia and Dangshe Ma, *Chem. Rev.*, 1992, **93**, 1137-1156.
2. G. Chopin, J. Rydberg and J. O. Liljenzin, *Radiochemistry and Nuclear Chemistry*, Butterworth-Heineman, Oxford, 1995, pp. 84-88.
3. R. D. Neirinckx, L. R. Canning, I. M. Piper, D. P. Nowotnik *et al.*, *J. Nucl. Med.*,1987, **28**, 191-202.
4. M. S. George, H. A. Ring, D. C. Costa, P. J. Ell, K. Kouris and P. H. Jarritt, *Neuroactivation and Neuroimaging with SPET*, Springer-Verlag, London, 1991, pp. 11-21.
5. D. E. Troutner, W. A. Volkert, T. J. Hoffman, R. A. Holmes, *Int. J. Appl. Radiat. Isot.*, 1984, **35**, 467-470.
6. S. Holm, A. R. Andersen, S. Vorstrup, N. A. Lassen, O. B. Paulson, and R. A. Holmes, *J. Nucl. Med.*, 1985, **26**, 1129-1134.
7. R. D. Neirinckx, J. F. Burke, R. C. Harrison, A. M. Forster, A. R. Andersen and N. A. Lassen, *J. Cereb. Blood Flow Metab.*, 1988, **8(Suppl. 1)**, S4-S12.
8. D. C. Costa, I. M. J. Motteux and A. C. McCready, *Eur. J. Nucl. Med.*, 1991, **18**, 503-506.
9. G. L. Kedderis and G. T. Miwa, *Drug Metab. Rev.*, 1988, **19**, 33-62.
10. K. E. Linder, Y. W. Chan, J. E. Cyr, M F Malley, D. P. Nowotnik, and A. D. Nunn, *J. Med. Chem.*, 1994, **37**, 9-17.
11. W. L Rumsey, B. Kuczynski, B. Patel *et al.*, *J. Nucl. Med.*, 1995, **36**, 1445-1450.
12. S. Jurisson, K. Aston, C. K. Fair, E. O. Schlemper, P. R. Sharp and D. Troutner, *Inorg. Chem.*, 1987, **26**, 3576-3582.
13. G. Brauers, C. M. Archer and J. F. Burke, manuscript in preparation.
14. C. M. Archer, B. Edwards, J. D. Kelly, A. C. King, J. F. Burke and A. L. M. Riley, in: *Technetium and Rhenium in Chemistry and Nuclear Medicine*, **4**, Ed. M. Nicolini, G. Bandoli and U. Mazzi, S. G. Editoriali, Padova, 1995, pp. 535-539.
15. D. F. Paulson, *J. Urol.*, 1979, **121**, 300-302.
16. G. M. Blake, M. A. Zivanovic, A. J. McEwan, D. M. Ackery , *Eur. J. Nucl. Med.*, 1986, **12**, 447-454.
17. Data on file at Amersham International.
18. V. J. Lewington, A. J. McEwan, D. M. Ackery, R. J. Bayley, D. H. Keeling, P. M. Macleod, A. T. Porter and M. A. Zivanovic, *Eur. J. Cancer*, 1991, **27**, 954-958.

